# Fructose 1,6-Bisphosphatase 2 Plays a Crucial Role in the Induction and Maintenance of Long-Term Potentiation

**DOI:** 10.3390/cells9061375

**Published:** 2020-06-01

**Authors:** Przemysław Duda, Tomasz Wójtowicz, Jakub Janczara, Daniel Krowarsch, Aleksandra Czyrek, Agnieszka Gizak, Dariusz Rakus

**Affiliations:** 1 Department of Molecular Physiology and Neurobiology, University of Wrocław, 50-335 Wrocław, Poland; przemyslaw.duda@uwr.edu.pl (P.D.); jakub.janczara@upwr.edu.pl (J.J.); agnieszka.gizak@uwr.edu.pl (A.G.); 2 Laboratory of Cell Biophysics, Nencki Institute of Experimental Biology, Polish Academy of Sciences, 02-093 Warsaw, Poland; t.wojtowicz@nencki.edu.pl; 3 Department of Biochemistry and Molecular Biology, Wrocław University of Environmental and Life Sciences, 50-375 Wrocław, Poland; 4 Department of Protein Biotechnology, University of Wrocław, 50-383 Wrocław, Poland; daniel.krowarsch@uwr.edu.pl (D.K.); aleksandra.czyrek@uwr.edu.pl (A.C.)

**Keywords:** memory formation, moonlighting protein, protein–protein interaction, astrocyte-neuron lactate shuttle

## Abstract

Long-term potentiation (LTP) is a molecular basis of memory formation. Here, we demonstrate that LTP critically depends on fructose 1,6-bisphosphatase 2 (Fbp2)—a glyconeogenic enzyme and moonlighting protein protecting mitochondria against stress. We show that LTP induction regulates Fbp2 association with neuronal mitochondria and Camk2 and that the Fbp2–Camk2 interaction correlates with Camk2 autophosphorylation. Silencing of Fbp2 expression or simultaneous inhibition and tetramerization of the enzyme with a synthetic effector mimicking the action of physiological inhibitors (NAD^+^ and AMP) abolishes Camk2 autoactivation and blocks formation of the early phase of LTP and expression of the late phase LTP markers. Astrocyte-derived lactate reduces NAD^+^/NADH ratio in neurons and thus diminishes the pool of tetrameric and increases the fraction of dimeric Fbp2. We therefore hypothesize that this NAD^+^-level-dependent increase of the Fbp2 dimer/tetramer ratio might be a crucial mechanism in which astrocyte–neuron lactate shuttle stimulates LTP formation.

## 1. Introduction

Long-term potentiation (LTP) in the hippocampus is the most studied example of synaptic plasticity, and it is regarded as the neuronal basis of learning [1].

Molecular events underlying the early phase of LTP include glutamate-stimulated Ca^2+^ influx into a neuron through N-methyl-D-aspartate receptors (NMDAR), subsequent activation of calcium-calmodulin-dependent protein kinase II (Camk2), and Camk2-dependent insertion of α-amino-3-hydroxy-5-methylisoxazole-4-propionate receptors (AMPAR) into the post-synaptic membrane [2,3].

The late phase of LTP, which encompasses changes lasting from minutes to hours, includes an induction of expression of a group of immediate early genes such as c-Fos, c-Jun and Arc which participate in different physiological processes associated with synaptic plasticity [4,5].

Although the role of crucial proteins, such as NMDAR, AMPAR and Camk2, and the significance of the astrocyte-derived lactate [6] in the LTP formation have been well established, the precise mechanism underlying the persistent synaptic enhancement is not fully understood.

In this paper, we present a line of evidence that fructose 1,6-bisphosphatase 2 (Fbp2) plays a crucial role in the induction and maintenance of LTP.

Fbp2 and the second isozyme of fructose 1,6-bisphosphatase, Fbp1, are regulators of gluconeogenesis. However, Fbp2 plays non-enzymatic functions in a cell due to its interactions with a variety of proteins [7,8]. Fbp2 protects mitochondria against the effects of stress stimuli such as elevated calcium levels resulting in mitochondrial swelling and decreased ATP synthesis [7]. Fbp2 also undergoes cytoplasmic–nuclear shuttling in hormone-dependent manners [9], and it has been recently demonstrated that nuclear Fbp2 regulates mitochondrial biogenesis via interaction with c-Myc [10]. 

Crystallographic studies have revealed that both the Fbp isozymes adopt a tetrameric quaternary structure [11]. Nonetheless, it has been shown that in solution, Fbp2 adopts various oligomeric states and that the dimeric Fbp2 is the main form interacting with mitochondria [12]. It has been demonstrated that the Fbp2 oligomerization is regulated by allosteric effectors (AMP and NAD^+^), which are indicators of energy and redox states of a cell [13]. Interestingly, it has been shown that oxidation of astrocyte-derived lactate in neurons contributes to memory formation [6] via elevation of NADH/NAD^+^ ratio [14]. 

Although neurons are not considered to synthesize glucose or glycogen from non-carbohydrates, the expression of both Fbp isozymes has been detected in vertebrate neurons [15]. Intriguingly, the neuronal enzyme is located in dendritic spines and synapses in close vicinity to mitochondria [16]. Unfortunately, because of a lack of isoform-specific antibodies, it is not possible to ultimately conclude which isoform localizes within synaptic terminals.

In the current study, we seek to investigate whether neuronal Fbp function is associated with regulation of mitochondrial polarity and whether it can affect synaptic plasticity in hippocampal neurons. 

The results presented here demonstrate for the first time that Fbp2 is an inherent element of the machinery of memory formation in the hippocampal structures. We demonstrate that hippocampal neurons express predominantly the Fbp2 isoform and that its colocalization with mitochondria is associated with the LTP induction. Silencing of Fbp2 expression or inhibition/tetramerization of the enzyme decreases mitochondrial polarity and blocks the LTP formation. Moreover, upon the LTP induction, Fbp2 interacts with Camk2 and stimulates its autophosphorylation, which is an indispensable step leading to formation of the early phase of the LTP. Intriguingly, the Fbp2 silencing/inhibition also blocks nuclear accumulation of Camk4 and abolishes the expression of LTP late phase markers such as c-Fos and c-Jun.

The capability of Fbp2 to adopt various quaternary conformations in the presence of AMP and NAD^+^, and hence its ability to interact with different cellular binding partners suggests that Fbp2 may be a decision center linking neurotransmitter signaling with energetic and redox state of brain during formation of memory traces.

## 2. Materials and Methods

### 2.1. Cell Culture and LTP Induction on Cultured Cells

All the procedures were approved by the Local Ethical Commission, and every effort was made to minimize the number of animals used in the experiments. 

Hippocampal neurons were isolated from newborn C57BL6 mice and cultured as described previously [17] with a minor modification—the glucose concentration in the culture medium was 2.5 mM. Neurons were seeded at a density of 25,000 cells/cm^2^ for in situ hybridization and immunofluorescent experiments and at a density of 50,000 cells/cm^2^ for homogenization or mitochondria isolation. All the experiments were performed using 14-day-old neuronal cultures. 

Prior to the LTP induction, the cultures were transferred to the Ringer’s solution (37 °C), and LTP was induced according to protocol described previously [18,19], with 1 μM strychnine and 200 μM glycine.

### 2.2. Immunofluorescence

The immunofluorescence studies were performed as described previously [20]. The cells were incubated overnight at 4 °C with respective primary antibodies: rabbit anti-Fbp (1:500, isolated and purified as described in [21]), mouse anti-Fbp (1:500, Santa Cruz Biotechnology, Dallas, TX, USA, sc-271799), rabbit anti-Camk2α (1:500, Merck KGaA, Darmstadt, Germany, C6974), mouse anti-Camk2α (1:500, ThermoFisher Scientific, Waltham, MA, USA, MA1-048), mouse anti-Camk2α pT286 (1:300, Abcam, Cambridge, UK, ab171095), rabbit anti-Camk4 (1:500, Merck KGaA, Darmstadt, Germany, HPA017206), mouse anti-Gsk3β pS9 (1:300, Merck KGaA, Darmstadt, Germany, 05-643), mouse anti-c-Fos (1:500, ThermoFisher Scientific, Waltham, MA, USA, MA1-21190), rabbit anti-c-Jun (1:500, ThermoFisher Scientific, Waltham, MA, USA, 702170), rabbit anti-Map2 (1:500, Merck KGaA, Darmstadt, Germany, M3696) and mouse anti-Map2 (1:500, Merck KGaA, Darmstadt, Germany, M4403). The primary antibodies were detected using fluorophore-labeled secondary antibodies: goat anti-mouse-AlexaFluor488 (1:1000; Abcam, Cambridge, UK, ab150113), goat anti-mouse-AlexaFluor633 (1:1000; ThermoFisher Scientific, Waltham, MA, USA, a21050), goat anti-rabbit-AlexaFluor 488 (1:1000; ThermoFisher Scientific, Waltham, MA, USA, a11034) or goat anti-rabbit-AlexaFluor633 (1:1000; ThermoFisher Scientific, Waltham, MA, USA, a21070). In controls, the primary antibodies were omitted. Nuclei were counterstained with DAPI. To visualize mitochondria, the cultures were incubated with 0.1 μM MitoTrackerTM Deep Red FM (ThermoFisher Scientific, Waltham, MA, USA, M22426) or with 2 µg/mL JC-1 Dye (ThermoFisher Scientific, Waltham, MA, USA, T3168) for 30 min at 37 °C, prior to the LTP induction.

### 2.3. Fluorescent In Situ Hybridization (FISH)

FISH was performed as described previously [20]. The following oligonucleotides complementary to mouse mRNA sequences were used: Fbp1 (5′-(Cyanine5)GACGGGTCCA GCATGAAGCA GTTGACACCA CAATCC-3′), Fbp2 (5′-(Cyanine3)GCACACAGCT GAGATACTCT TGCACATCCT CAGGGGAC-3′). All the oligonucleotides were synthesized by Merck KGaA. In controls, the oligonucleotide probes were omitted.

### 2.4. Inhibitors

The following inhibitors were used: 5 μM or 10 μM Fbp inhibitor (5-chloro-2-(N-(2,5-dichlorobenzenesulfonamido))-benzoxazole; Cayman Chemicals, Ann Arbor, MI, USA, 18860), 25 μM MCU inhibitor (Ru360; Merck KGaA, Darmstadt, Germany, 557440), 5 μM SERCA inhibitor (thapsigargin; Merck KGaA, Darmstadt, Germany, T9033). Each of the inhibitors was added to neuronal culture 30 min prior to the LTP induction.

### 2.5. Fbp2 Expression Silencing

**Figure 1:**
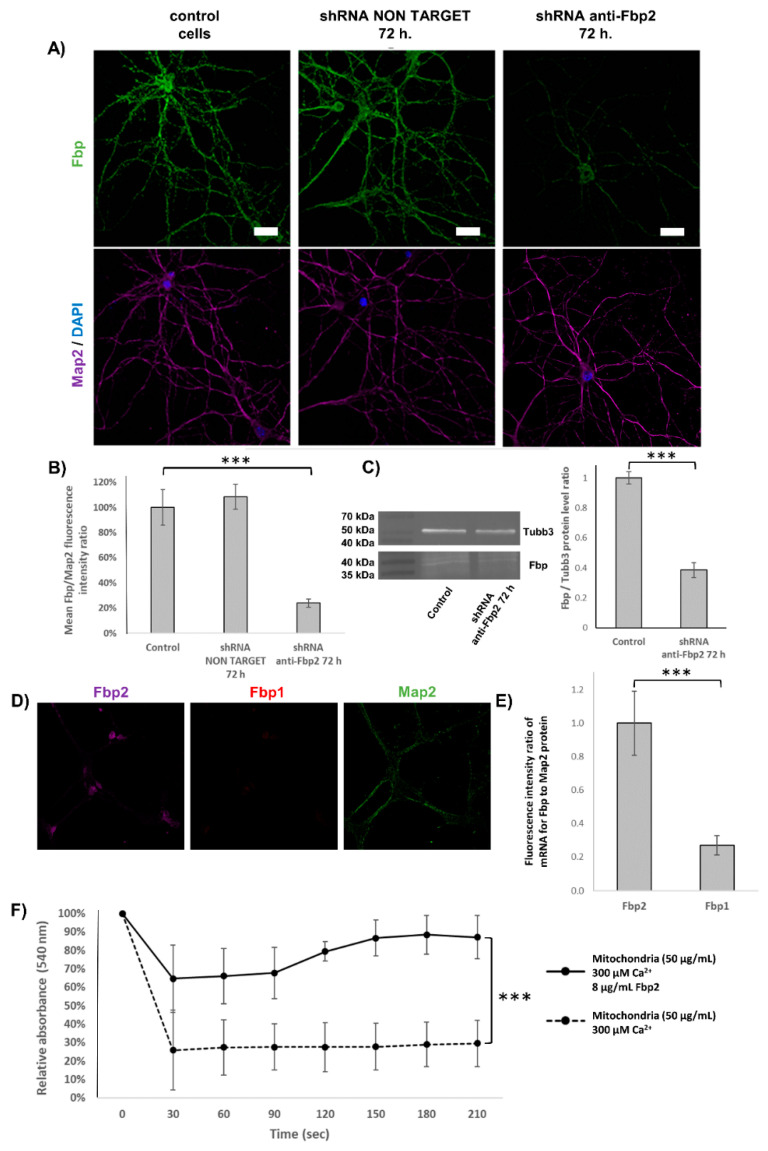
(**A**–**C**) Silencing of fructose 1,6-bisphosphatase 2 (Fbp2) expression in cultured hippocampal neurons. (**A**) Expression of Fbp in hippocampal neurons: untreated (left image), treated with antisense shRNA (middle image) and shRNA against Fbp2 (right image). Bar = 20 µm, N = 30 regions of interest (ROIs). (**B**) Decrease in Fbp2-associated fluorescence (expressed as the ratio of Fbp2 to Map2 fluorescence). (**C**) Western blot analysis of Fbp2-silenced hippocampal neurons and densitometric analysis of the protein level. Tubulin β3 was used as a reference protein. (**D**,**E**) Fbp2 is the main isoform of Fbp in hippocampal neurons. (**D**) In situ hybridization demonstrates that fluorescent signal associated with mRNA for Fbp2 is much stronger than for Fbp1. (**E**) Quantification of the mRNA for Fbp2 and Fbp1. Bar = 20 µm, N = 30 ROIs. (**F**) Fbp2 protects neuronal mitochondria against calcium-induced swelling. The amount of swollen mitochondria is inversely proportional to the decrease of relative absorbance at 540 nm. The absorbance measured before Ca^2+^ addition was assumed to be 100%. The values are given as a mean and SD. *** indicates *p* < 0.001.

Fbp2 expression was silenced with shRNA against 3′UTR of Fbp2 mRNA (FBP2 MISSION^TM^ shRNA Lentiviral Transduction Particles; Merck KGaA, Darmstadt, Germany, SHCLNV-NM_007994). The shRNA sequence was 5′-CCGGAGATGA ATGAGCTATG GAGATCTCGA GATCTCCATA GCTCATTCAT CTTTTTTG-3′. The transduction particles were added to neuronal cultures in the amount of 2.5 × 10^5^, and the transduction was carried out for 72 h. In controls, the cells were transduced with the non-target shRNA (MISSION^TM^ TRC2 pLKO.5 Non-Target shRNA Control Plasmid DNA) at the concentration of 5 ng/mL. The silencing was monitored by immunofluorescent microscopy and WB analysis (Figure 1A–C).

### 2.6. Co-Immunoprecipitation

In the co-immunoprecipitation experiment, recombinant human Fbp2 (purified as described by [12]) and Camk2α (ThermoFisher Scientific, Waltham, MA, USA, PR4586C) proteins were used. Briefly, Camk2α and Fbp2 (final concentrations each of the proteins were 0.37 µM) were mixed and incubated overnight at 4 °C. Next, the mixture was incubated overnight at 4 °C with 50 µg/mL of antibodies against Fbp or Camk2α, and the complexes were precipitated using 200 µL/mL of the Protein G Agarose (Merck). The precipitates were centrifuged at 4000× *g* for 2 min and washed with PBS. In control reactions, the precipitating antibodies were omitted. The precipitates were then resuspended in the Laemmli’s buffer and resolved by SDS–PAGE, and Western Blot analyses were performed with the use of primary antibodies detecting Fbp when the precipitate was obtained with anti-Camk2α antibodies, and detecting Camk2α when the precipitate was obtained using anti-Fbp antibodies.

### 2.7. Preparation of Acute Brain Slices

Brain slices (350 µm thick) were prepared from C57BL6 mice aged P30-P90 as described in [22]. The slices were cut with McIlwain Tissue Chopper (Ted Pella, Inc. Redding, CA, USA).

### 2.8. Electrophysiological Recordings

Field recordings (fEPSPs) were performed as described previously [22,23]. All drugs were bath-applied, and all recordings were made in CA1 stratum radiatum (150–200 μm from the stratum pyramidale) in ACSF perfused at 7 mL/min. Schaffer-collateral axons were stimulated with a concentric bipolar electrode (0.1 Hz, 0.25 ms) while fEPSPs were recorded with glass micropipettes filled with ACSF (1–3 MΩ resistance). Input–output (I–O) relationships were built for fEPSP amplitudes upon monotonically increased stimuli in the range of 0–300 μA (16 points, applied once at 0.1 Hz). Baseline stimulation was set at 0.1 Hz, and the stimulation strength was set to 40% of the maximum fEPSP. Synaptic potentiation was evoked with tetanic stimulation, HFS (4 × 100 Hz, 1 s duration, with 10 s inter-train intervals) following 15 min of baseline recording.

Recordings of synaptic currents were performed in cultured primary hippocampal neurons, in voltage clamp mode of the patch-clamp technique, as described earlier [24], with modifications. Briefly, holding potential was set at −60 mV, and spontaneous excitatory postsynaptic currents (sEPSCs) were recorded in the Ringer’s solution, in the presence of strychnine (1 µM) and 5 mM glucose. NMDAR-dependent synaptic potentiation was evoked by bath application of Ringer’s solution containing reduced magnesium concentration (0.5 mM), 100 µM glycine and 30 mM glucose [18]. sEPSCs were recorded in 20 s sweeps and alteration in sEPSCs frequency, duration, and amplitude was expressed as relative change in average sEPSC area per sweep. All control recordings were made in the presence of drug diluents. All electrophysiological data were analyzed in pClamp 10 (Molecular Devices, LLC, San Jose, CA, USA) software package.

### 2.9. Biolayer Interferometry

Measurements of the kinetics of Fbp2–Camk2a interaction was performed using ForteBio Octet K2 (Pall ForteBio, Fremont, CA, USA) and high-specificity anti-His antibody biosensor (His2, Pall ForteBio, Fremont, CA). Studies were performed at 25 °C with shaking at 1000 rpm in PBS supplemented with 2 mM Mg^2+^ and 10 μM Ca^2+^. Sensor tips were hydrated in buffer for 30 min prior to use. The 96-microwell plates were filled with 200 μL of buffer or samples and incubated for 10 min prior measurements for system stabilization. Camk2α (3.5 μg/mL) was loaded on the His2 sensor for 120 s and washed for 60 s. A reference sensor without Camk2α served as a background control. Association and dissociation phases (300 s each) were monitored at various concentrations of Fbp2 protein ranging from 50 to 400 nM. Kinetic parameters were determined by global fitting with the 1:1 model. Response values from the last 10 s of the association phase were averaged and used for equilibrium dissociation constants calculation. Data were analyzed with ForteBio Data Analysis 11.0 software (Pall ForteBio, San Jose, CA, USA).

### 2.10. Thermophoresis

Fbp2–Camk2α interaction was studied using microscale thermophoresis with the NanoTemper Monolith NT.115 instrument (NanoTemper Technologies GmbH, Munich, Germany). Camk2a was labeled with the Monolith His-Tag Labeling Kit RED tris-NTA 2nd Generation (Nanotemper Technologies GmbH, Munich, Germany) according to the manufacturer’s instruction. Various concentrations of Fbp2 (0.397 nM–13 µM) were titrated against labeled Camk2α (50 nM) in PBS buffer, supplemented with 0.05% Tween. Samples were loaded into the Premium Coated Capillaries (NanoTemper Technologies GmbH, Munich, Germany), and thermophoresis was measured using 80% LED power and 40% infrared laser power in the ambient temperature of 23 °C. F0 and F1 times were −1–0 s and 1.1–2.1 s, respectively. Datasets were processed with the MO.Affinity Analysis software (NanoTemper Technologies GmbH, Munich, Germany).

### 2.11. In Situ Detection of Protein Interaction

Endogenous detection of Fbp-Camk2α interaction was performed using the Duolink^®^ In Situ Orange Starter Kit Mouse/Rabbit (Merck KGaA, Darmstadt, Germany) according to the protocol provided by the manufacturer, using mouse anti-Fbp (1:500, Santa Cruz Biotechnology, Dallas, TX, USA, sc-271799) and rabbit anti-Camk2α (1:500, Merck KGaA, Darmstadt, Germany, C6974) primary antibodies. In controls, the primary antibodies were omitted.

### 2.12. Western Blot

Western blot of neuronal protein extracts was performed as described in [20] using the following antibodies: rabbit anti-Fbp (1:2000, [21]), mouse anti-Camk2α (1:1000, ThermoFisher Scientific, Waltham, MA, USA, MA1-048) and rabbit anti-tubulin β3 (1:1000, Synaptic System GmbH, Göttingen, Germany, 302302). Goat anti-rabbit peroxidase-conjugated (1:50,000, Merck KGaA, Darmstadt, Germany, a0545) and goat anti-mouse peroxidase-conjugated antibodies (1:50,000, Merck KGaA, Darmstadt, Germany, a9044) were used as secondary antibodies, and the reaction was visualized using the SuperSignal^TM^ West Pico PLUS Chemiluminescent Substrate (ThermoFisher Scientific, Waltham, MA, USA).

### 2.13. Isolation of Mitochondria and Measurement of Mitochondrial Swelling and Polarization

The neuronal mitochondria were purified on sucrose gradient, and swelling of the organelles was monitored as an abrupt decrease in light absorbance at 540 nm as described in [7]. To determine the polarization of the organelles, the JC-1 Dye (ThermoFisher Scientific, Waltham, MA, USA, T3168) was used. The experiment was performed as described in [12]. The data were expressed as a ratio of JC-1 fluorescence intensities at 590 and 530 nm. 

### 2.14. Confocal Microscopy and Fluorescence Analysis

Confocal microscopy analysis was performed as described previously [20]. The quantification of the fluorescence was carried out using the ImageJ software [25]. For the analysis of protein and mRNA expression, the mean fluorescence intensity was measured from regions of interest (ROIs). The ROIs were defined based on the Map2 immunostaining, which delineates the neuronal cells. The mean Camk4 fluorescence intensity was measured in the areas of ROIs, or in the nuclei area of Map2-positive cells, and was normalized as a ratio relative to the mean intensity of Map2 staining in the same ROIs [26]. In the immunodetection and in situ hybridization experiments, N stands for number of ROIs analyzed. 

For the analysis of Fbp–mitochondria colocalization, the Manders’ coefficient was used. The coefficient varies from 0 (no colocalization) to 1 (100% of colocalization). It was determined as the ratio of summed intensities of pixels from the Fbp image for which the intensity in the mitochondrial channel was above zero to the total intensity in the Fbp channel [27].

### 2.15. Statistical Analysis

Data were analyzed using SigmaPlot 11 (Systat Software, San Jose, CA, USA) and Microsoft Excel 2016 software. Results are expressed as a mean and standard deviation. If not stated otherwise, data were checked for normality using the Shapiro–Wilk test, and for the evaluation of statistical significance, the two-tailed Student’s T-test or one- or two-way ANOVA was used. A probability of *p* < 0.05 was considered to represent a significant difference. All the experiments were done at least in triplicate. 

## 3. Results

### 3.1. Fbp2 Colocalization with Neuronal Mitochondria

**Figure 2:**
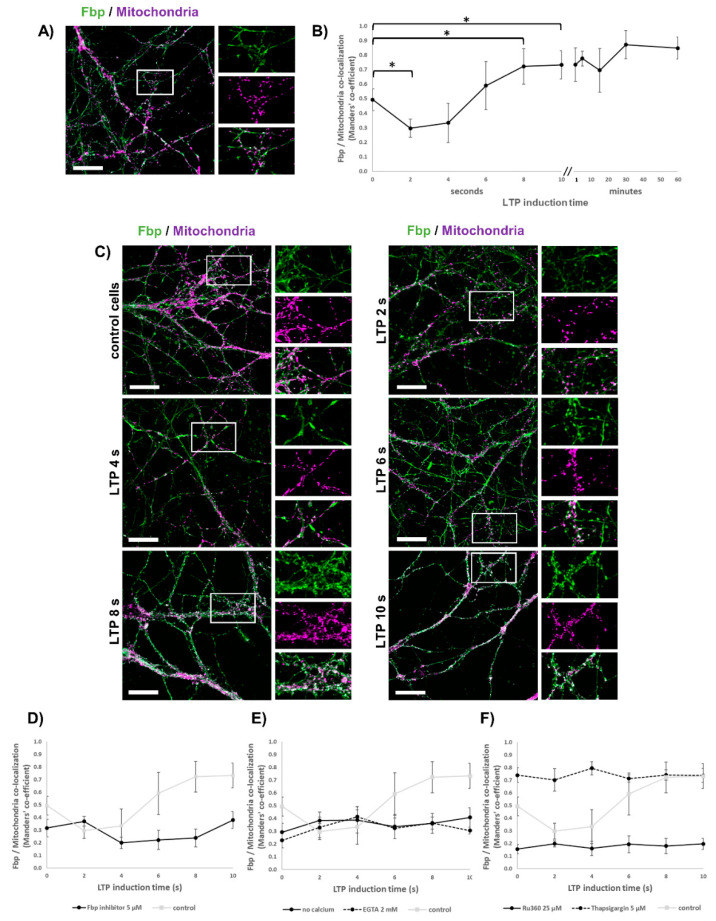
Fbp colocalizes with neuronal mitochondria in a calcium-dependent manner. (**A**,**B**) In control (unstimulated) neurons, about half of Fbp molecules colocalize with mitochondria. (**B**,**C**) Stimulation of long-term potentiation (LTP) decreases the colocalization in the first 4 s, but subsequently, the colocalization gradually increases again. (**D**) Fbp inhibition/tetramerization decreases colocalization of the enzyme with mitochondria both in unstimulated and in stimulated neurons. (**E**) Inhibition (with Ru360) of Ca^2+^ uptake by mitochondria results in decreased Fbp–mitochondria colocalization while block (using thapsigargin) of Ca^2+^ uptake to endoplasmic reticulum results in increase of the colocalization. (**F**) Absence of Ca^2+^ in the extracellular solution or chelation of the cations decreases Fbp–mitochondria colocalization both in unstimulated and in stimulated neurons. Bar = 10 μm. The values are given as a mean and SD. * indicates *p* < 0.05. For each time point, the colocalization was measured from 40 images.

To explore the potential involvement of Fbp in regulation of neuronal mitochondria polarization and synaptic plasticity, we used cultured mouse hippocampal neurons and immunofluorescent techniques. In control conditions, about half of the Fbp-associated fluorescent signal colocalized with mitochondrial staining (Figure 2A,B). Because neurons may express both Fbp isoforms [15], we assessed the ratio of Fbp2 to Fbp1 using in situ hybridization technique, which revealed that Fbp2 accounted for approximately 75% of the total neuronal Fbp (Figure 1D,E). Since our previous study has demonstrated that Fbp2, but not Fbp1, interacts with and protects cardiomyocytic mitochondria against swelling [28] and since Fbp2 silencing did not influence expression of mRNA for Fbp1 (data not shown), it can be assumed that practically all changes in the localization of Fbp shown in this study were related to Fbp2.

Chemical induction of LTP stimulated time-dependent changes in the colocalization of the Fbp2- and mitochondria-related signals (Figure 2B,C). During the first 4 s after the beginning of the LTP induction, we observed a significant decrease of the Fbp signal associated with mitochondria (Figure 2B,C), but in the next 4 s, the Fbp–mitochondria colocalization increased by approx. 60% and persisted for the next 60 min (Figure 2B,C).

LTP is initiated by Ca^2+^ influx through NMDARs [29]. It has been demonstrated that calcium ions must first enter mitochondria in order to activate Camk2 in a ROS- and Ca^2+^-dependent manner [30,31]. On the other hand, a prolonged, modestly elevated level of cytosolic Ca^2+^ inhibits LTP formation [32]. Since it has been demonstrated that Fbp2 binds to cardiomyocyte mitochondria under Gsk3β inhibition or elevation of cellular Ca^2+^ levels [7] we tested whether and which calcium stores can regulate interaction of neuronal Fbp2 with mitochondria. 

As could be expected, the presence of calcium in the extracellular medium was a prerequisite to observe the LTP-related changes in Fbp2–mitochondria colocalization (Figure 2E) and absence of Ca^2+^ in the extracellular solution, or chelation of the cation with EGTA (Figure 2E) resulted in much weaker interaction of the enzyme with mitochondria. 

In the presence of Ca^2+^ in the external solution, the inhibition of mitochondrial calcium uptake with Ru360 [33] was also associated with a very low level of the Fbp–mitochondria colocalization and induction of LTP in such conditions had no effect on the subcellular distribution of the enzyme (Figure 2F). On the other hand, inhibition of sarco/endoplasmic reticulum Ca^2+^-ATPase (SERCA) with thapsigargin, which is known to elevate concentration of cytosolic Ca^2+^ [34], strongly stimulated the Fbp2 binding to mitochondria (Figure 2F). Since it is well established that the lack of calcium in the extracellular fluid or application of Ru360 or thapsigargin inhibits LTP formation [35,36,37], our results suggest that mitochondrial Ca^2+^ uptake correlates with the Fbp2 binding to the organelles; however, such an association is not sufficient for the LTP formation.

**Figure 3:**
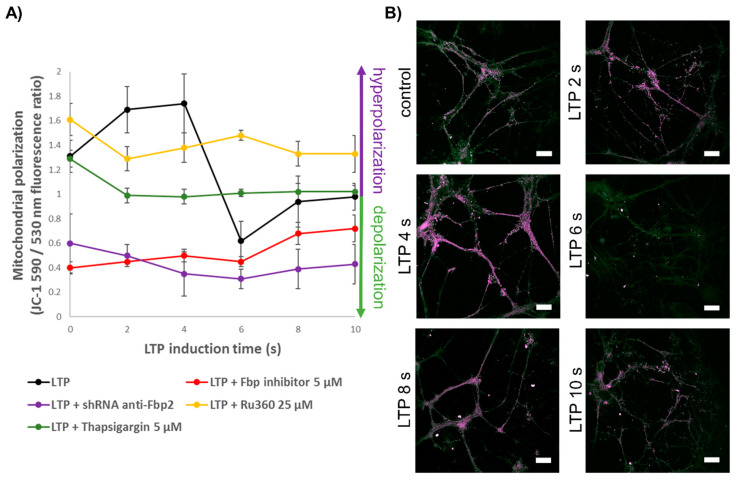
LTP-associated changes in mitochondrial membrane polarization. (**A**) Induction of LTP results in a short event of mitochondrial hyperpolarization followed by a period of their depolarization. Inhibition/tetramerization of Fbp (red line) as well as its silencing (blue line) results in depolarization of mitochondria. In the presence of thapsigargin (an inhibitor of the sarco/endoplasmic reticulum Ca^2+^-ATPase; green line) or Ru360 (an inhibitor of Ca^2+^ uptake to mitochondria; yellow line), mitochondria are weakly hyperpolarized and depolarized, respectively, as compared to basal polarization (at time 0). Except in the control conditions (no inhibition/tetramerization or silencing of Fbp; black line), the induction of LTP does not affect mitochondrial polarization. (**B**) Time-dependent changes in mitochondrial polarization after the LTP induction. Bar = 20 μm. The values are given as a mean and SD. N = 30 ROIs.

Basically, induction of LTP results in a long-lasting mitochondria depolarization [38] which is associated with a phasic occurrence of mitoflashes, transient events of higher mitochondrial activity comprising mitochondrial depolarization and reactive oxygen species production [39]. Results presented here demonstrated, however, that mitochondria underwent hyperpolarization during the first 4 s after beginning of the LTP induction (Figure 3A,B). Such a short (<4 s) and transient hyperpolarization of mitochondria upon the LTP induction/glutamate stimulation has never been observed earlier. It presumably reflects a transient elevation of cytosolic Ca^2+^ concentration after influx of the ions via NMDAR but before their buffering by mitochondria. It has been previously shown that in neurons treated with 0.1 mM NMDA, the increases in cytosolic and in mitochondrial Ca^2+^ were virtually identical [40]. However, we did not use any exogenous NMDAR agonist to induce LTP [18,19], so the opening of NMDARs was mediated only by endogenous glutamate, the titer of which was presumably many times lower than the concentration of NMDA used in the previous study. Thus, it is likely that in our experimental model, the rate of Ca^2+^ influx into cytosol via NMDAR was lower and there was a lag in the mitochondrial cation uptake, which in turn induced a transient mitochondrial membrane hyperpolarization event. 

Importantly, we observed that during the hyperpolarization event (Figure 3A,B), the colocalization of Fbp2 with mitochondria was significantly decreased (Figure 2B). Four sec after the beginning of the LTP induction, we could see a fast decrease in the polarization (Figure 3A,B), and it was synchronized in time with an increased association of Fbp2 with mitochondria (Figure 2B).

A reciprocal correlation between the Fbp2–mitochondria colocalization and mitochondrial membrane potential was also observed when neurons were treated with Ru360 (Figure 2F and Figure 3A). On the other hand, in the presence of thapsigargin, the association of Fbp2 with the organelles was significantly elevated but the mitochondrial polarity was unaffected (Figure 2F and Figure 3A).

In the presence of both inhibitors, the induction of LTP did not change the basal Fbp2 association with mitochondria and the mitochondrial polarization was only moderately decreased (Figure 2E and Figure 3A).

Our previous experiments have demonstrated that Fbp2 not only associates with mitochondria in a Ca^2+^-dependent manner but that it also protects them against calcium-induced swelling [7], and basically, we observed the same effect studying mitochondria from the hippocampal neurons culture (Figure 1F). Since we have also shown that dimeric Fbp2 is the preferred oligomeric form of the enzyme for interactions with mitochondria [12], we asked how oligomerization of Fbp2 affects mitochondria polarization in a resting state and after the chemical LTP induction. Because binding of allosteric inhibitors, such as AMP and NAD^+^, to Fbp induces and/or stabilizes the tetrameric conformation of the enzyme [11,41], we used a synthetic Fbp inhibitor, 5-chloro-2-(N-(2,5-dichlorobenzenesulfonamido))-benzoxazole (further called iFbp), which mimics the action of the physiological allosteric inhibitors [42,43].

After incubation of neurons with iFbp, we observed a low level of the Fbp2–mitochondria colocalization which correlated with a strong depolarization of mitochondrial membrane. Both phenomena were not affected by the LTP induction (Figure 2D and Figure 3A). Similarly strong depolarization of mitochondria was observed after partial silencing of Fbp2 expression with siRNA (Figure 3A). This reinforces the hypothesis that the Fbp2 interaction with mitochondria is essential for the proper polarization of the organelles in neurons, both during resting state and after LTP induction.

### 3.2. The Effect of Fbp2 on LTP Formation

**Figure 4:**
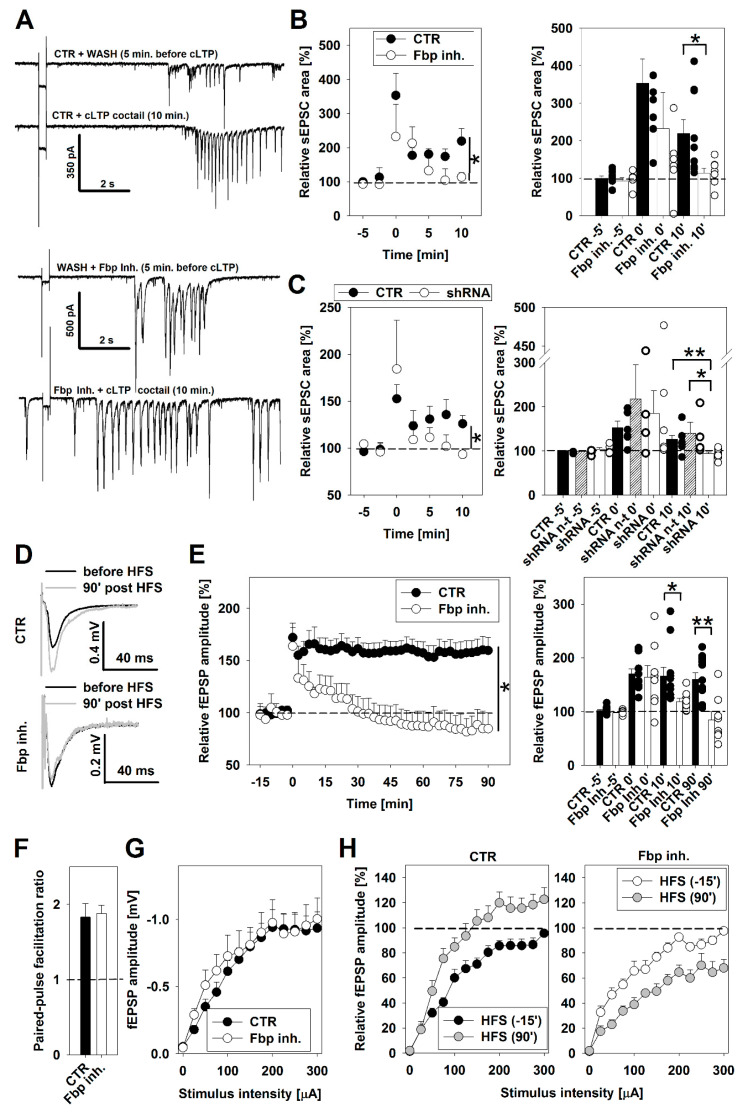
Impact of Fbp2 on synaptic potentiation of excitatory synapses. (**A**) Exemplary traces of spontaneous excitatory postsynaptic currents (sEPSCs) recorded in hippocampal neuronal cultures. sEPSCs were recorded continuously before (“WASH”) and for 10 min following pharmacological induction of LTP. The upregulation of frequency and amplitude of sEPSCs is more pronounced in control conditions (upper panel), as compared to that observed in the presence of Fbp inhibitor (lower panel). (**B**) Time course (left panel) and relative sEPSC area change before (−5 min) and up to 10 min post LTP induction (right panel) in control neurons (black) and neurons incubated with the Fbp inhibitor (white). In the presence of the inhibitor, potentiation of sEPSC is significantly reduced at 10 min time point (n = 9 and 7 neurons per group, respectively, N = 3 cultures, *p* < 0.05; unpaired Student’s T-test). (**C**) Time course (left panel) and relative sEPSC area change before (−5 min) and up to 10 min post LTP induction (right panel) in control neurons (black), neurons transduced with a non-target shRNA (dashed white) and neurons transduced with shRNA against Fbp2 (white). The silencing of Fbp2 abolished enhancement of sEPSC following LTP as compared to the control group and shRNA non-target group (one-way ANOVA with Holm-Sidak post-hoc, N = 3 cultures, F_(2,14)_ = 3.5; *p* < 0.05). (**D**) Exemplary traces of fEPSPs evoked in mouse acute hippocampal slices, at CA1 stratum radiatum in response to stimulation of Schaffer collaterals, before (black traces) and 1.5 h post-high-frequency stimulation (HFS, grey traces). Note that in contrast to control slices, presence of the Fbp inhibitor (10 µM) abolished enhancement of fEPSPs post-HFS. (**E**) The time course (left panel) and average fEPSP amplitudes recorded before (−5 min) and up to 90 min post-HFS in control slices (black) or following incubation with the Fbp inhibitor throughout recording (white). Note that the inhibitor significantly abolished LTP magnitude (n = 11 and 8 slices, and N = 8 and 6 mice per group, respectively; *p* < 0.01, unpaired Student’s T-test). (**F**) Average paired-pulse facilitation ratio recorded in response to paired (50 ms gap) stimuli, in control slices (black) and those treated with Fbp2 inhibitor for 15 min (white) did not significantly differ (n = 7 slices per group, N = 5 animals, *p* = 0.88, Student’s unpaired T-test). (**G**) Input–output curves obtained in response to monotonically increasing stimuli (0–300 uA) recorded in control slices (black) and those incubated with Fbp2 inhibitor for 15 min (white). The inhibitor did not affect the input–output curves recorded before LTP induction (F_(2,244)_ = 0.02, *p* = 0.88, two-way ANOVA). (**H**) In control slices (left panel), LTP induction with HFS resulted in a significant leftward shift in the input–output curves recorded 90 min later (HFS 90’) compared to 15 min before HFS (−15 min’, F_(2,140)_ = 86.88, n = 11 slices, N = 8 animals, *p* < 0.01, two-way ANOVA). In contrast, in slices incubated with Fbp2 inhibitor (right panel), a significant rightward shift was detected at HFS 90’ (F_(2,101)_ = 117.83, n = 8 slices, N = 6 animals, *p* < 0.01, two-way ANOVA). Asterisks indicate significant difference. Error bars indicate SEM.

Fbp2 silencing or inhibition/tetramerization significantly decreased neuronal mitochondria polarization and made it insensitive to LTP induction. Thus, we checked whether the manipulation with Fbp2 quaternary structure (using iFbp) or concentration (siRNA-mediated silencing) may affect functional aspects of synaptic potentiation in excitatory synapses. Firstly, we analyzed spontaneous excitatory postsynaptic currents (sEPSCs) in cultured hippocampal neurons. The Fbp2 inhibitor did not significantly affect the baseline sEPSC area (−19.51 ± 5.45 and −28.01 ± 10.26 pA *x* ms for control cultures and those treated with Fbp2 inhibitor, respectively; n = 9 and n = 7 neurons, respectively; *p* = 0.45; see also Figure 4B). Upon pharmacological induction of NMDAR-dependent synaptic potentiation in control conditions, a robust upregulation of sEPSC amplitude and frequency and an enlarged average sEPSC area were observed (Figure 4A,B). In contrast, when iFbp (5 µM) was applied, despite initial sEPSC enlargement, LTP was significantly impaired from the 10th minute (Figure 4A,B). 

In another set of experiments, we silenced Fbp2 expression in hippocampal neurons. Here, the baseline sEPSC area was −45 ± 7.8, −60 ± 19 and −56 ± 10.5 pA *x* ms for control, non-target sequence shRNA group and Fbp2 shRNA group, respectively (n = 6, 4 and 7 neurons, respectively; F_(2,14)_ = 0.293, *p* = 0.75, one-way ANOVA). As shown in Figure 4C, in control neurons, the LTP induction resulted in an upregulation of sEPSCs. However, when Fbp2 expression was silenced, an initial sEPSCs enhancement was observed at time 0, but it was eventually abolished in the next 10 min (Figure 4C).

To determine if Fbp2 is required for LTP in the hippocampal network, we recorded field excitatory postsynaptic potentials (fEPSPs) in the CA1 region of hippocampal brain slices. Signals recorded in control slices in response to basal stimulation (applied at 0.1 Hz) had the amplitude of −0.38 ± 0.05 mV (n = 11 slices, N = 8 animals) and did not differ upon application of Fbp2 inhibitor (−0.42 ± 0.10 mV, n = 11 and 8 slices, respectively; *p* = 0.70, Student’s unpaired T-test). Next, we induced LTP with high-frequency stimulation (HFS, 4 × 100 Hz). We found that in control conditions, HFS resulted in a robust and stable LTP in excitatory synapses over a period of 90 min (Figure 4D,E). The application of iFbp had no impact on basal fEPSPs (Figure 4E). However, the electrically induced LTP was significantly impaired from the time point of 10 min and was completely abolished in the presence of iFbp (Figure 4D,E). To determine whether the observed effects may be due to altered short-term plasticity and presynaptic release, we recorded fEPSPs in response to paired stimulation (50 ms gap between stimuli). We found no difference in paired-pulse facilitation ratio (Figure 4F; *p* = 0.88, Student’s unpaired T-test). We also recorded fEPSPs in response to a wide range of monotonically increased stimuli. As shown in Figure 4G, input–output curves recorded before HFS in control slices, and those incubated with Fbp2 inhibitor for 15 min, did not significantly differ (F_(2,244)_ = 0.02, p = 0.88, two-way ANOVA). However, the input–output relationship looked strikingly different 90 min post-HFS. Notably, in control slices, LTP resulted in a significant leftward shift in the input–output curves recorded 90 min later (Figure 4H; F_(2,140)_ = 86.88, n = 11 slices, N = 8 animals, *p* < 0.01, two-way ANOVA). In contrast, in slices incubated with Fbp2 inhibitor (10 µM), a significant rightward shift was detected 90 min post HFS (F_(2,101)_ = 117.83, n = 8 slices, N = 6 animals, *p* < 0.01, two-way ANOVA). Altogether, Fbp2 inhibitor did not affect basal synaptic transmission, synaptic responses to a wide range of stimuli or short-term synaptic plasticity. However, the presence of Fbp2 is critical to support NMDAR-dependent functional scaling of excitatory synapses both in the early and in the late phase.

Afterwards, we verified the electrophysiological results by monitoring changes in fluorescence signals associated with protein markers of both LTP phases.

To monitor the induction of the early phase, we checked phosphorylation status of proteins crucial for the formation of LTP, such as Camk2 and AMPA receptor (AMPAR). Autophosphorylation leading to autoactivation of Camk2 (Camk2α pT286) is considered one of the first events in the canonical pathway of memory formation [44]. Phosphorylation of AMPAR subunits by Camk2 stimulates excitatory synaptic plasticity by incorporation of the receptor into the postsynaptic membrane [45]. We also searched for the presence of the inhibitory phosphorylation of Gsk3β (Gsk3β pS9), the kinase which must be inhibited by autoactivated Camk2 during the excitatory potentiation [46,47].

**Figure 5:**
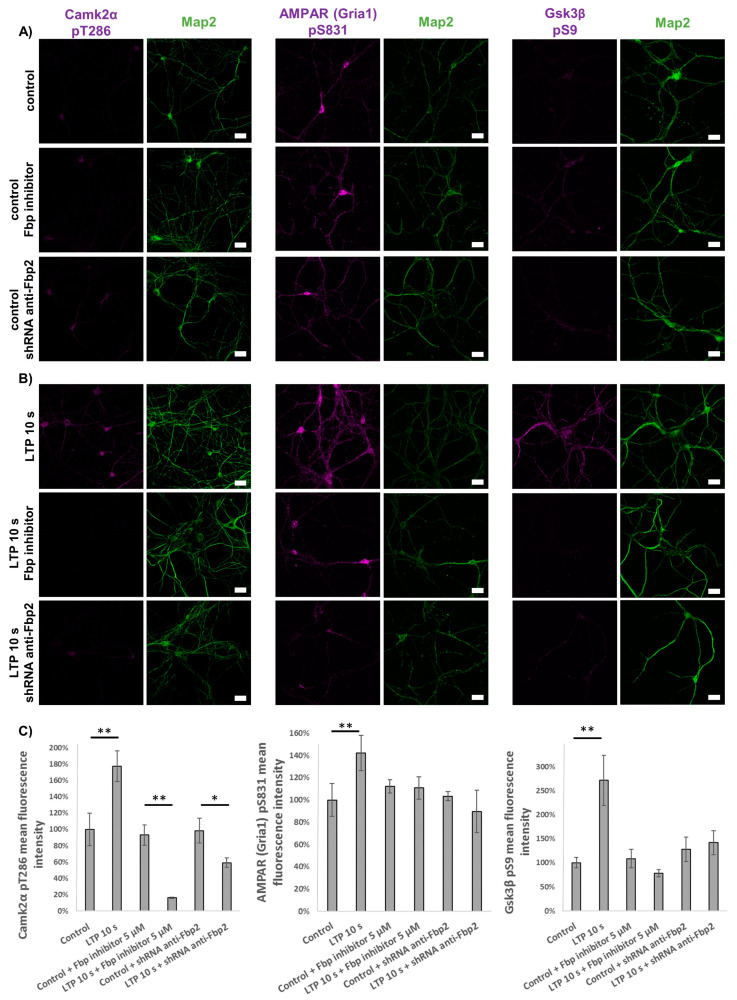
Silencing or inhibition/tetramerization of Fbp2 block expression of the early phase of LTP. (**A**) Fluorescence signals associated with phosphorylated forms of Camk2α, AMPAR (Gria1) and Gsk3β are not affected by Fbp2 silencing or inhibition/tetramerization in neurons in which LTP was not induced. (**B**) Induction of LTP stimulates phosphorylation of Camk2α, AMPAR and Gsk3β (upper row) but manipulation with Fbp2 amount and structure strongly reduces the phosphorylation. (**C**) Quantification of the fluorescence associated with phosphorylated forms of Camk2α (pT286), AMPAR (pS831) and Gsk3β (pS9) as compared to the fluorescence recorded in unstimulated neurons (controls). Bar = 20 μm. For each condition, N = 25 ROIs. The values are given as a mean and SD. * indicates *p* < 0.05, ** indicates *p* < 0.01.

Under resting conditions, we observed that both silencing and inhibition/tetramerization of Fbp2 had no effect on phosphorylation status of the studied proteins (Figure 5A). The induction of LTP resulted in a significant increase of the level of phosphorylation of Camk2α, AMPAR and Gsk3β, indicating postsynaptic potentiation (Figure 5B–E).

However, both silencing and inhibition of Fbp2 almost completely blocked the phosphorylation of all the monitored proteins (Figure 5B–E), and thus the early phase of LTP.

Since the electrophysiological studies demonstrated that silencing of Fbp2 inhibited the LTP formation (Figure 4), we addressed the question of whether Fbp2 defects are reflected by deficits in the expression of the late LTP markers. 

**Figure 6:**
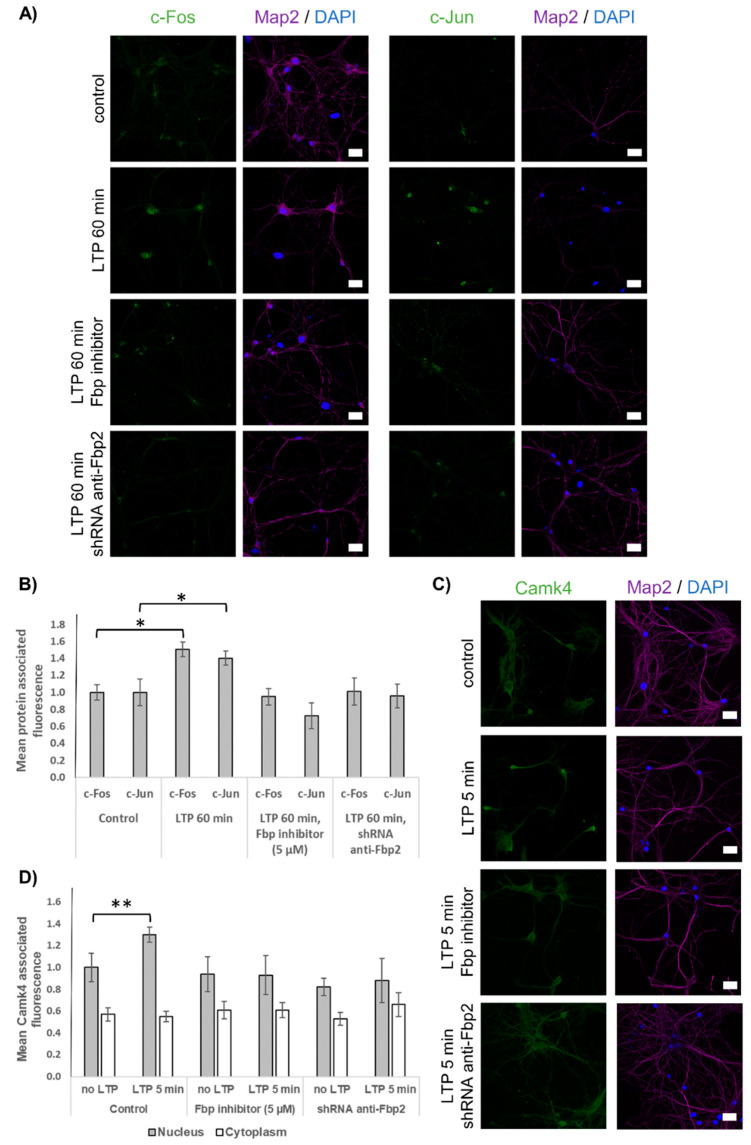
Expression of the late-phase LTP depends on Fbp2 structure and concentration. (**A**) Induction of LTP increases fluorescence related to c-Fos (left panel) and c-Jun (right panel); however, inhibition of Fbp or reduced concentration of the enzyme abolishes the expression of both the transcription factors. (**B**) Quantification of mean c-Fos- and c-Jun-associated fluorescence in neurons after the LTP induction as compared to the fluorescence in neurons in which LTP was not induced. For each condition, N = 30 ROIs. (**C**) Nuclear translocation of Camk4 is blocked by Fbp inhibition/tetramerization or silencing. (**D**) Mean Camk4-associated fluorescence from neuronal nuclei after the LTP induction as compared with that observed in unstimulated neurons (control). For each condition, N = 20 ROIs. Bar = 20 μm. The values are given as a mean and SD. * indicates *p* < 0.05, ** indicates *p* < 0.01.

We tested the expression of c-Jun and c-Fos, two transcriptional factors responsible for the formation of LTP [5] and nuclear localization of Camk4, a kinase involved in stimulation of the expression of LTP-associated transcriptional factors [48]. Our results revealed that both inhibition/tetramerization and silencing of Fbp2 almost completely abolished LTP-induced expression of the transcription factors (Figure 6A) and blocked the LTP-dependent nuclear accumulation of Camk4 (Figure 6B). This corroborates the results obtained using electrophysiological methods (Figure 4).

### 3.3. Fbp2 Interaction with Camk2

Both inhibition/tetramerization and silencing of Fbp2 blocked Camk2 autoactivation during the LTP induction (Figure 5B) and led to disruption of the LTP formation (Figure 4, Figure 5B–E and Figure 6). It raised an intriguing question if Fbp2 is directly involved, via protein–protein interactions, in the regulation of Camk2 autophosphorylation.

**Figure 7:**
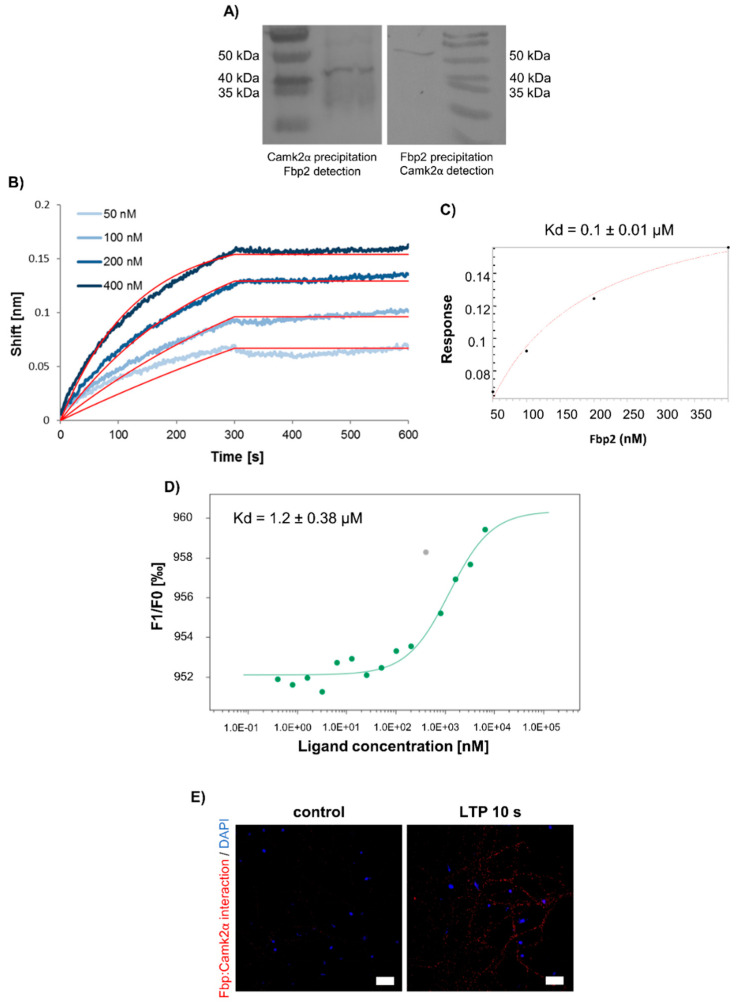
Fbp2 interacts with Camk2α. (**A**) Co-immunoprecipitation of Fbp2–Camk2α complex; the left image shows Western blot detection of Fbp2 after precipitation of the complex with anti-Camk2α antibodies, while the right shows immunodetection of Camk2α after the precipitation with the use of anti-Fbp IgG. (**B**) Biolayer interferometry (BLI) sensograms of Fbp2 binding to Camk2α. The red lines represent global fit to the 1:1 interaction model. (**C**) Steady-state analysis of the Fbp2–Camk2α interaction. (**D**) Fbp2 binds to Camk2α (Kd = 1.2 µM) as quantified using microscale thermophoresis experiments. (**E**) In situ detection of Fbp2 interaction with Camk2α in 10 s after the LTP induction. Red spots indicate the places in which the enzymes interact with each other (Bar = 20 μm).

The results of our co-immunoprecipitation experiment with the use of purified proteins demonstrated that Camk2α and Fbp2 may associate with each other (Figure 7A). Since immunoprecipitation is a semiquantitative technique, we used biolayer interferometry (BLI) and thermophoresis to describe the Fbp2–Camk2α interaction.

Using the BLI, we observed a very strong interaction between Fbp2 and Camk2α, with picomolar apparent binding constant (Figure 7B). Unexpectedly, after withdrawal of the free (unbound) Fbp2 from the assay medium, we were not able to detect any dissociation of Fbp2 from Camk2α for over five minutes (Figure 7B). It may confirm the significant strength of the interaction; however, it may also reflect global and long-lasting structural changes within the Camk2α-Fbp2 complex. Additionally, we performed steady-state analysis based on the recorded data. The dissociation constant calculated from the fitted saturation binding curves equaled to 0.1 µM (Figure 7C).

In the BLI method, Camk2α was covalently attached to the sensor. Thus, to check if both proteins can interact with each other in solution, we used the thermophoresis- (also called thermodiffusion-) based technique. The results revealed that Fbp2 binds to Camk2α with dissociation constant KD = 1.2 µM (Figure 7D).

Finally, using the Duolink technique, we verified the proteins’ interaction in cultured neurons. In control conditions, we found only a low degree of colocalization of Fbp2 and Camk2α (Figure 7E). However, the chemical induction of LTP resulted in a mass appearance of fluorescent signal associated with the Fbp2–Camk2α complex (Figure 7E), corroborating the interaction of the proteins in living cells and its LTP-induction dependence.

## 4. Discussion

LTP and long-term synaptic depression (LTD) are regarded as the basic mechanism of long-term memory formation. Here, we present several lines of evidence that the presence of Fbp2 is indispensable for the induction and expression of LTP.

Fbp2 is a glyconeogenic enzyme. However, neurons are not supposed to synthesize glycogen from carbohydrate precursors [49]. Moreover, expression of Fbp2 as well as its liver isoform—Fbp1—in neurons is very low. Hence, it is not likely that the enzyme may significantly contribute to any metabolic pathway. Nevertheless, Fbp2 is also involved in protection of mitochondria against calcium-induced stress in a non-enzymatic manner [7]. Since neuronal mitochondria are continuously exposed to elevated Ca^2+^ levels during activation of the cells, the association of Fbp2 with these organelles may be a mechanism protecting the organelles and whole neurons against defective-mitochondria-mediated death.

In this report, we demonstrated that about half of the Fbp2 molecules are colocalized with mitochondria in unstimulated neurons and that induction of LTP results in dynamic, Ca^2+^- and mitochondrial-membrane-potential-dependent changes in the degree of the colocalization. Generally, except for the short early phase of the LTP formation (the first ~8 s), a majority of the enzyme is associated with mitochondria for at least 60 min—a period in which mitochondria are moderately depolarized with a phasic occurrence of mitoflashes—and the expression of proteins involved in the late phase of LTP is stimulated. Such a high level of the colocalization, even in unstimulated neurons, is in line with the protective role of Fbp2 in mitochondria subjected to stress stimuli.

Importantly, we also show that the presence of Fbp in neurons is required for initiation of the LTP formation. Fbp2 interacts with Camk2α during NMDAR-dependent activity at excitatory synapses and stimulates Camk2 phosphorylation, which is the first step in the LTP induction. Silencing of Fbp2 results in disruption of both the early phase of LTP (as determined by changes in phosphorylation status of Camk2, AMPAR and Gsk3) and the late phase of the memory formation (monitored by expression of such LTP-associated transcription factors as c-Fos and c-Jun and nuclear localization of Camk4). 

Interestingly, the application of iFbp reproduces the effect of the enzyme silencing on the LTP formation. In a cell, Fbp2 exists in equilibrium of various oligomeric forms, predominantly dimers and tetramers [12], and binding of physiological allosteric inhibitors, such as AMP and NAD^+^, stabilizes the tetrameric conformation and inhibits enzymatic activity of Fbp2 [12]. Since iFbp mimics the effect of the endogenous inhibitors, it may be presumed that Fbp2 adopts the tetrameric quaternary conformation in cells treated with this compound. Thus, it could be inferred that the dimeric form of Fbp2 is the preferred form of the enzyme, which interacts with Camk2α and ensures the LTP formation. 

The preferred binding of the dimeric Fbp2 to mitochondria and Camk2 may explain the mechanism of astrocyte–neuron lactate shuttle (ANLS) stimulation of LTP induction. ANLS denotes the process in which astrocyte-derived lactate is converted by neurons to pyruvate and used to fulfill energy requirements of neuronal cells [50,51]. Although the significance of ANLS for the whole brain energetics is under dispute [52,53,54,55], the absolute requirement for the lactate transport from astrocytes to neurons during LTP formation is not controversial [6,55,56]. It has been demonstrated that the astrocyte-derived lactate fuels expression and translation of memory-formation-associated mRNA [14,56]. Moreover, it has been shown that elevation of NADH/NAD^+^ ratio, which accompanies oxidation of lactate to pyruvate, stimulates activity of NMDAR by modifying neuronal redox state [14]. Importantly, the reduced NAD+/NADH ratio is also a factor increasing the pool of dimeric Fbp2 because NAD^+^ stimulates the enzyme tetramerization and inactivation [12]. Thus, the uptake of astrocyte-derived lactate by neurons might be considered one of the mechanisms stimulating Fbp2 dimerization in neurons and hence enabling—via Camk2 activation—induction of LTP and protecting mitochondria against calcium stress. 

Concluding, data presented here demonstrate that induction and formation of the NMDAR-dependent plasticity in hippocampal neurons and slices depends on the presence of Fbp2. Neighboring astrocytes may participate in the induction of LTP in the ANLS-dependent manner via regulation of the oligomeric state of Fbp2 in neurons. 

Our findings open a new area of study directly linking memory formation to energy state of the brain. Further analysis of the interdependence between Fbp2 oligomerization state and interactions of the enzyme with cellular binding partners in the context of astrocyte–neuron cross-talk is likely to reveal a precise mechanistic insight into synaptic plasticity.

The results presented here also suggest that a decreased binding of Fbp2 to mitochondria caused by a reduced amount of the enzyme or presence of defective forms of Fbp2 (with impaired ability of oligomerization or reduced affinity to mitochondria) might be associated with certain mitochondrial function-related brain disorders [57]. 

Additionally, the proper mode of Fbp2 interaction with mitochondria depends also on Gsk3β inactivation. Gsk3β is a kinase whose overactivity is attributed to Alzheimer’s disease pathogenesis [58]. Thus, an attractive hypothesis is that disruption of mitochondrial function during progression of the disease [59] at least partially results from the inability of Fbp2 to protect mitochondria against stress stimuli in the presence of permanently active Gsk3β.

Finally, it is essentially unknown whether in neurons, Fbp2 expression and the enzyme post-translational modifications change during aging and whether such changes impact brain plasticity and lead to age-dependent memory decline.
